# Enhancing Ovarian Tumor Diagnosis: Performance of Convolutional Neural Networks in Classifying Ovarian Masses Using Ultrasound Images

**DOI:** 10.3390/jcm13144123

**Published:** 2024-07-15

**Authors:** Maria Giourga, Ioannis Petropoulos, Sofoklis Stavros, Anastasios Potiris, Angeliki Gerede, Ioakeim Sapantzoglou, Maria Fanaki, Eleni Papamattheou, Christina Karasmani, Theodoros Karampitsakos, Spyridon Topis, Athanasios Zikopoulos, Georgios Daskalakis, Ekaterini Domali

**Affiliations:** 1 1st Department of Obstetrics and Gynecology, National and Kapodistrian University of Athens, 11528 Athens, Greece; kimsap1990@hotmail.com (I.S.); maria.fanaki@gmail.com (M.F.); eleni.papamattheou@gmail.com (E.P.); ckarasmani@gmail.com (C.K.); gdaskalakis@yahoo.com (G.D.); kdomali@yahoo.fr (E.D.); 2 School of Electrical & Computer Engineering, National Technical University of Athens, 15772 Athens, Greece; 3 Third Department of Obstetrics and Gynecology, University Hospital “ATTIKON”, Medical School of the National and Kapodistrian University of Athens, 12462 Athens, Greece; sfstavrou@med.uoa.gr (S.S.); apotiris@med.uoa.gr (A.P.); theokarampitsakos@hotmail.com (T.K.); spyros.topis1996@gmail.com (S.T.); thanzik92@gmail.com (A.Z.); 4 Department of Obstetrics and Gynecology, University of Thrace, 68100 Alexandroupolis, Greece; agerede@otenet.gr

**Keywords:** ultrasonography, deep learning, ovarian cancer, artificial intelligence, convolutional neural network, diagnosis

## Abstract

**Background/Objectives:** This study aims to create a strong binary classifier and evaluate the performance of pre-trained convolutional neural networks (CNNs) to effectively distinguish between benign and malignant ovarian tumors from still ultrasound images. **Methods:** The dataset consisted of 3510 ultrasound images from 585 women with ovarian tumors, 390 benign and 195 malignant, that were classified by experts and verified by histopathology. A 20% to80% split for training and validation was applied within a k-fold cross-validation framework, ensuring comprehensive utilization of the dataset. The final classifier was an aggregate of three pre-trained CNNs (VGG16, ResNet50, and InceptionNet), with experimentation focusing on the aggregation weights and decision threshold probability for the classification of each mass. **Results:** The aggregate model outperformed all individual models, achieving an average sensitivity of 96.5% and specificity of 88.1% compared to the subjective assessment’s (SA) 95.9% sensitivity and 93.9% specificity. All the above results were calculated at a decision threshold probability of 0.2. Notably, misclassifications made by the model were similar to those made by SA. **Conclusions:** CNNs and AI-assisted image analysis can enhance the diagnosis and aid ultrasonographers with less experience by minimizing errors. Further research is needed to fine-tune CNNs and validate their performance in diverse clinical settings, potentially leading to even higher sensitivity and overall accuracy.

## 1. Introduction

Ovarian cancer is one of the leading causes of cancer-related deaths among women worldwide. It is calculated as the deadliest gynecological cancer with the highest morbidity and mortality, accounting for more than 12,700 deaths each year [1]. It is often called a silent killer due to the lack of symptoms, especially in early stages, leading to late diagnosis, thus limiting treatment options [2]. This highlights the importance of early detection and immediate referral of such patients in tertiary oncology centers [3]. The most effective and easily accessible tool for the categorization of ovarian masses is ultrasonography [4]. Subjective assessment (SA) by expert ultrasonographers achieves the highest sensitivity and specificity rates, although there is a shortage of experts in this field [5]. Proposed models, such as Data System Ultrasound (ORADS-US), and models of the International Ovarian Tumor Analysis (IOTA), such as Simple Rules Risk (SRR), Risk of Malignancy Algorithm (RMI), logistic regression models LR1 and LR2, and Assessment of Different NEoplasias in the adneXa (ADNEX model), are widely used internationally and can achieve high rates of overall accuracy [6,7,8,9,10,11]. Although these tools have been validated externally and can achieve comparable results with subjective assessment, their performance depends on the examiner [4,12]. This fact underscores the critical role of human expertise in achieving the optimal diagnostic accuracy, though the risk of human error remains. According to published and ongoing research, an addition to the medical field is the use of artificial intelligence and deep learning, which has the potential to enhance diagnostic precision, decision-making, and limit human error [13,14,15,16]. Convolutional neural networks (CNNs), a subset of deep learning algorithms, offer automated image analysis that can extract complex features and patterns of medical images, such as X-rays, MRI, CT, and ultrasound images [17,18,19].

Researchers have already utilized pre-trained CNNs and reported promising results equivalent to experienced sonographers’ subjective assessment [14,17,20,21,22,23,24]. However, using neural networks in clinical practice necessitates substantial validation and the creation of large datasets of annotated ovarian images that can be utilized to train the models appropriately. The fusion of CNNs and medical imaging has the potential to transform everyday clinical practice and ovarian tumor diagnosis [13].

## 2. Materials and Methods

### 2.1. Dataset

We utilized a comprehensive dataset comprising still ultrasound images from patients with ovarian tumors treated in our tertiary gynecological oncology center from 2011 to 2023. The dataset consisted of 8324 collected ultrasound images from 585 women with ovarian tumors. Out of these masses, 390 were benign, and 195 were malignant ovarian tumors. For training and testing the model, 3510 images were used in total by choosing 6 representative still ultrasound images from each patient. Out of these images, 539 had calipers. The cases were selected from our center archives to represent different histopathology types. There was a slight imbalance in our dataset since 66.7% of the tumors were benign while the remaining 33.3% were malignant. This difference is reasonable, as it depicts the actual probability of malignancy in patients presenting with ovarian cysts [25]. The explant pathology report was used as a reference diagnosis and the masses were classified according to the International Federation of Gynecology and Obstetrics (FIGO) classifications for ovarian cancer staging [26]. The histopathology of the ovarian masses used can be found in Table 1.

### 2.2. Ultrasound Assessment

Based on our center protocol, women with ovarian lesions undergo an ultrasound examination preoperatively. Patients presenting with ovarian tumors that underwent ultrasound assessment up to 120 days before surgery were eligible for inclusion in the study. All ultrasound examinations were performed according to IOTA rules and definitions by 5 individual gynecologists with 6 to 21 years of experience. All ultrasound examiners are IOTA-certified and they perform more than 600 ultrasound examinations per year. The images used in our study were collected by 6 different ultrasound machines (General Electric HealthCare LOGIQ P6, Voluson S6, S8, E6, E10, and Samsung HS40, Athens, Greece). All cases were prospectively classified by our experts, while being blind to histopathology reports. They categorized the masses as benign, malignant, and inconclusive.

### 2.3. Data Processing

After the extraction from the ultrasound system, the images were cropped to the region of interest (ROI). A combinational approach was employed for this task, which utilized both manual and auto-cropping. The simple algorithm that was developed, after deidentifying the images by removing edge areas, isolated the ovaries and excluded unwanted structures (uterus, cervix, bladder, etc.). The key idea was to use pixel value comparisons and assume most of the ultrasound images had similar structures, meaning that deviations from the expected structure would translate into unwanted regions that were removed. Although the results had to be manually reviewed afterwards, the margin of error was low enough to deem the algorithm effective. The input layer of each CNN model has a shape of (150, 150) and ReLU activation. The images were thus resized accordingly, into 150-by-150 pixel-sized square images. Since the original images were in RGB format (three color channels, size of x, y, 3), a flattened layer was also added before passing the images as input to the model. The pixel values were also normalized over the entire dataset. Data augmentation was employed to artificially increase the dataset size and prevent model overfitting. Several geometric transformations were applied, including image flipping, rotation, shearing, and zoom. The color properties of the images were also modified by using brightness and contrast adjustments. After experimentation, noise injection was not incorporated, as the results showed it hindered the overall system performance.

### 2.4. Model Building

The core principle we adhered to for building the CNN models was transfer learning. Three different pre-trained models from ImageNet were used as building blocks for our final classifier system, VGG16, Resnet50, and InceptionNet3. Each of the three models consisted of the pre-trained base, with the modified input layer mentioned above, followed by a flattened layer and two dense layers. The output of each model was a single neuron, representing the probability of malignancy, for each image passed as input. The final classifier for each image was an aggregate of the three different networks, essentially a weighted average of the three output probabilities. The exact value of the weights was one of the primary parameters of experimentation in our study. The criterion by which the final weights were chosen was maximizing the model’s accuracy. The values used in our study were 0.5, 0.25, and 0.25, for VGG15, Resnet50, and InceptionNet3, respectively. However, the weights were purposedly left as a tunable hyperparameter during the final system design. After every image was passed through the full model, the probabilities for the representative images of each patient were averaged out, and the final probability of malignancy was calculated. This value was then compared with the decision threshold and the case was finally labeled as benign or malignant. The exact value of the decision threshold was the second parameter greatly examined in this study. Similar to the weights, different decision thresholds can affect which type of cases were successfully labeled and which were not. The value of the decision threshold was 0.2. As with the case of the pre-trained model weights, the decision threshold is modifiable, according to the requirements and needs of different scenarios. A cross-validation approach based on the k-fold technique was employed to evaluate the performance of the CNN models. A 20% to 80% split was utilized for training and validation, ensuring comprehensive data utilization across multiple folds. The final classifier was constructed through a weighted aggregation of three pre-trained CNN architectures: VGG16, ResNet50, and InceptionNet. Parameters such as aggregation weights and decision threshold probability were fine-tuned to optimize classification performance.

### 2.5. Training Process

As stated before, each model initially had its input layer modified to a shape of 150-by-150 neurons with ReLU activation. After the convolutional base, a flattened layer consisting of 8192 neurons was added. Two dense layers followed, one with 256 neurons and ReLU activation, and the final layer of the model with a single neuron (representing the binary classifier decision) and sigmoid activation function. The convolutional base was trainable for all the training epochs and, therefore, the pre-trained weights served as a solid starting point. A batch size of 20 images was used, in conjunction with batch normalization. Binary cross-entropy was employed as the loss function while RMSprop (root mean squared propagation) was used for optimization, with an adaptive learning rate of 2x10^-5^. The training went on for 50 epochs each time, using callbacks and early stopping. The main python package used was Keras from TensorFlow and the entirety of the training process ran on an NVIDIA Quadro M2000 graphics card.

## 3. Results

**Figure 1:**
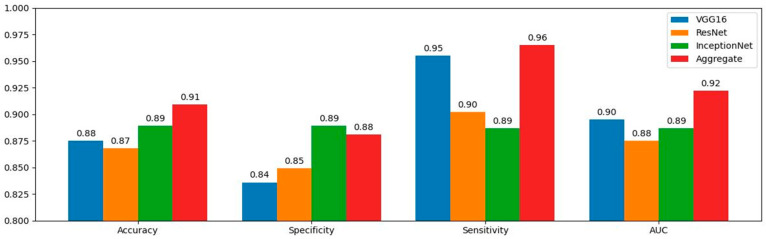
Performance metrics for diagnosing ovarian tumors as benign or malignant for the CNN models, VGG16 (-), ResNet50 (-), and InceptionNet (-), and the aggregate model (-).

**Figure 2:**
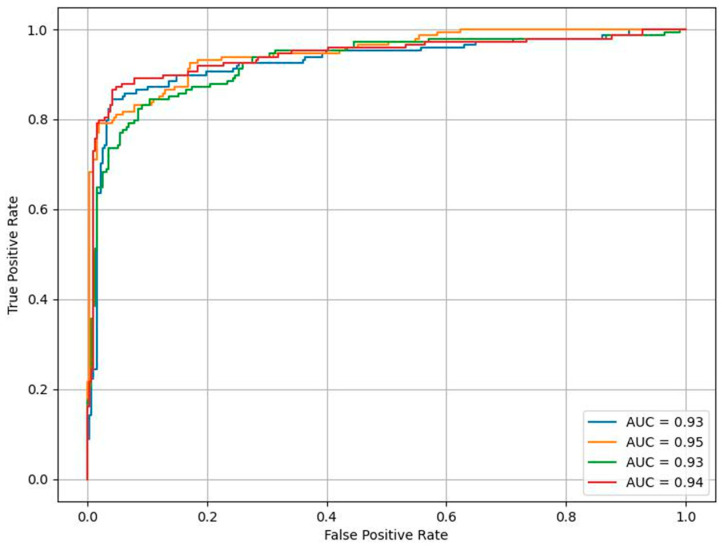
ROC curves for diagnosing ovarian tumors as benign or malignant for the CNN models, VGG16 (**-**), ResNet50 (**-**), and InceptionNet (**-**), and the aggregate model (**-**).

The performances of the three different CNN models (VGG16, ResNet50, and InceptionNet) and the aggregate model were evaluated and based on overall accuracy, sensitivity, specificity, and AUC, with their corresponding 95% confidence interval (CI). Bootstrapping was used to calculate the CI. The results can be found in Table 2 and Figure 1. The corresponding ROC curves appear in Figure 2.

VGG16 scored an overall accuracy of 87.50% averaged out over all the k-folds. It achieved an average sensitivity of 95.5% at a specificity of 83.60%. The best-performing fold had a sensitivity of 100%, while the worst-performing fold had a sensitivity of 90.5% with a specificity of 80.0%. It was the overall best-performing CNN among the three pre-trained models, while considering all the performance metrics. ResNet50 had an overall accuracy of 86.8%. The average sensitivity was 90.2% with specificity equal to 84.9%. Even though the highest-scoring fold reached an accuracy of 95.7%, ResNet50 had a bad-performing fold in terms of specificity (68.0%). It had the lowest overall performance among the pre-trained models, except for a marginally higher average specificity when compared to VGG16. InceptionNet achieved accuracy equal to 88.9%, a sensitivity of 88.7%, and a specificity of 88.9%. It is the only pre-trained model that had a higher average specificity than sensitivity, with the highest specificity among the three. It is essentially a “failsafe” in the aggregate model, increasing its ability to correctly label benign tumors and reduce false positives. The aggregate model outperformed all models, only being marginally second to InceptionNet in terms of specificity. It achieved an average sensitivity of 96.5% with a specificity of 88.1%, and the overall accuracy was 90.9%. All the above results were calculated at a decision threshold probability of 0.2.

**Figure 3:**
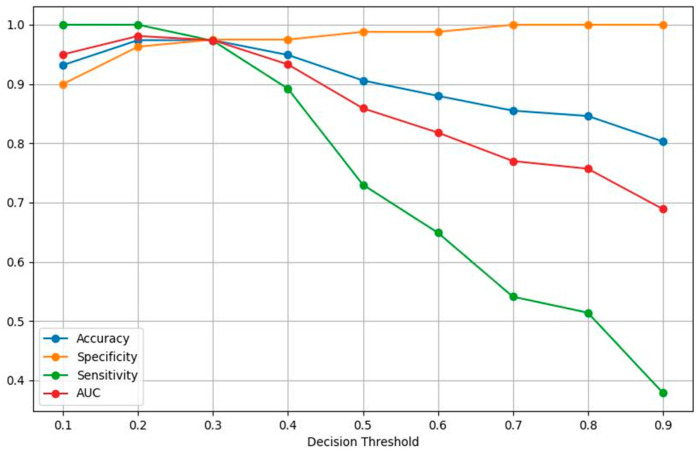
Performance metrics as a function of the decision threshold probability for the aggregate CNN model, the best-performing k-fold.

The constraint applied to choose a value for the decision threshold was to maximize sensitivity while also maintaining high specificity. After enforcing a requirement of having a minimum sensitivity of 98.0%, the value of 0.2 was chosen as the benchmark for all our evaluation runs. At the default decision value of 0.5, the specificity of the aggregate model increased significantly at 98.8%; however, the sensitivity dropped to a below acceptable value, considering the implications in a diagnosis of malignancy. Table 3 and Figure 3 portray the values of all the performance metrics of the aggregate model (for the best-performing k-fold) for decision thresholds between 0.1 and 0.9 with a step of 0.1, with a decision threshold probability of 0.2.

**Figure 4:**
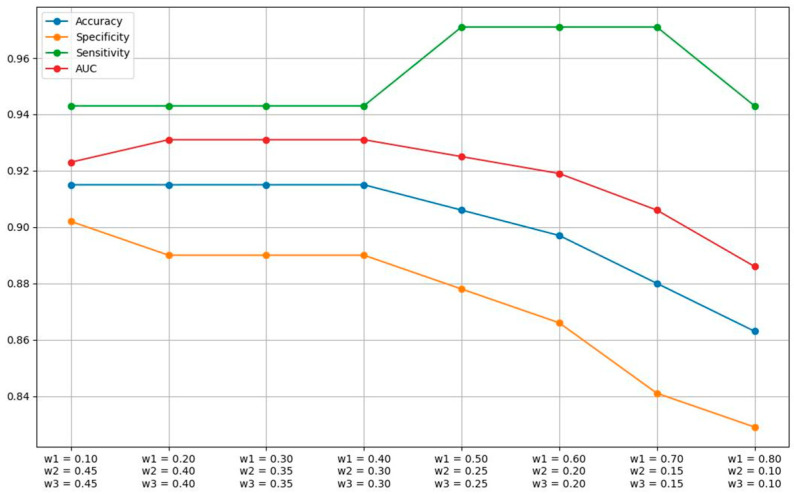
Graph of the performance metrics for the aggregate model in an average k-fold with a test-set size of 117 cases, for varying weights of each pre-trained model. Decision threshold of 0.2.

The same principle was applied when selecting the weights of the three pre-trained models in the aggregate network. As shown by our results, the best-performing model in terms of sensitivity and overall performance was VGG16. Even though InceptionNet performed worse than the other two models in terms of sensitivity, its presence is required in the aggregate model to ensure a high specificity. We executed several evaluation runs of the aggregate model, while modifying the weight of the VGG16 model and evenly splitting the remaining weight between the other two models. After experimenting with various values between 0.1 and 0.8, the optimal combination of weights according to our results was 0.5, 0.25, and 0.25, for VGG16, ResNet50, and InceptionNet, respectively. Table 4 and Figure 4 depict the performance metrics with adjusted weights for each model, for an average k-fold with a test size of 117 patients and decision threshold equal to 0.2.

The ultrasonographers achieved a sensitivity of 95.9%, a specificity of 93.6%, and an overall accuracy of 94.2% while classifying the masses. Out of 585 cases, 4 cases were classified as inconclusive by subjective assessment. Out of these, three were malignant according to the histopathology report, and one was benign. Cases classified as inconclusive by the ultrasonographers were categorized as malignant for the purpose of calculating the performance metrics.

**Figure 5:**
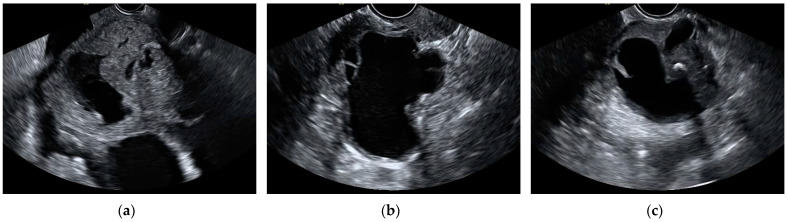
Still ultrasound images of malignant ovarian masses misclassified as benign by the aggregate model. (**a**) Endometrioid ovarian carcinoma, (**b**) high-grade serous ovarian carcinoma, and (**c**) high-grade serous ovarian carcinoma.

**Figure 6:**
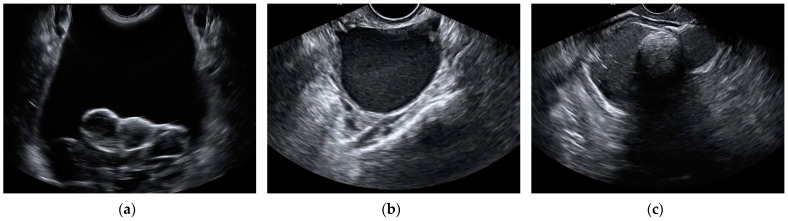
Still ultrasound images of benign ovarian masses misclassified as malignant by the aggregate model. (**a**) Serous cystadenoma, (**b**) endometrioma, and (**c**) mature cystic teratoma.

The most frequent histological type in false-positive cases by the aggregate model were cystadenomas and endometriomas. In total, 14 cystadenomas and 14 endometriomas were wrongfully classified as malignant by the model. Additionally, 10 cystadenomas and 5 endometriomas were also misclassified as malignant by subjective assessment. Out of these, eight cystadenomas and four endometriomas were misclassified by both the aggregate model and the SA. The total amount of mislabeled cases by the model was calculated as the sum of false positives and false negatives, added over all k-folds. The histopathology of misclassified cases by the aggregate model and subjective assessment can be found in Table 5. Ultrasound images of misclassified images by the CNNs can be found in Figure 5 and Figure 6.

## 4. Discussion

The use of AI in the evaluation of ultrasound images of ovarian masses seems to be promising. The first steps in assessing the risk of ovarian cancer using AI started as early as 1999 by various study groups [27,28,29,30]. By 2010, Lucidame et al. used HistoScanning, a technique based on the quantification of tissue disorganization in malignancies of backscattered ultrasound 3D raw volumes, achieving a sensitivity of 98% using a small dataset [31]. Multiple subsequent studies have demonstrated high levels of sensitivity specificity and overall accuracy, often surpassing the outcomes of subjective assessment [13,17,20,21,22,23,24,32,33,34].

Several recent studies in the field have also utilized radiomics combined with deep learning to develop a fused predictive model for malignancy in ovarian tumors [20,35,36,37,38]. The key procedure employed is image segmentation followed by feature extraction. The results are then propagated and used in the traditional deep learning techniques we followed in our study. Barcroft et al. used the Dice surface coefficient to measure the segmentation performance, achieving remarkable results [20]. Du et al. fed the extracted features into a logistic regression (LR) model and used a nomogram to evaluate and visualize their outcomes [36]. Despite promising results in the bibliography, we opted out of incorporating radiomics in our study for two main reasons. Firstly, it can severely increase the complexity and development time of the model. Secondly, it has been documented that combining radiomics with deep learning often leads to overfitting the model, due to specific features being included in the training [39].

Our aggregate model achieved comparable results to previously published studies, demonstrating a sensitivity of 96.5% and a specificity of 88.1%. Notably, our model’s sensitivity was higher than the SA in our dataset, which was 95.9%, though it exhibited lower specificity compared to SA (88.1% vs. 93.6%). When compared to the International Ovarian Tumor Analysis (IOTA) studies with a sensitivity of 90.4% and specificity of 92.7%, the SA indicated slightly higher performance metrics than the SA in our dataset [40].

Our findings align with those of Christiansen et al., who developed and trained deep neural networks, achieving a sensitivity of 97.1% and specificity of 89.3%, which were equivalent to subjective assessments using data from 785 ovarian masses obtained from two different centers [21]. Similarly, Hsu et al. reported a mean sensitivity and specificity of 91.37% and 92.92%, respectively, by combining different neural networks [23]. Gao et al., using a diverse dataset and two external validation sets, demonstrated that CNN models could significantly aid non-expert ultrasonographers, improving sensitivity by 12.3% (82.7% vs. 70.4%) and specificity by 8.6% (88.7% vs. 80.1%), underscoring the potential of AI-assisted ultrasonography to reduce human error and facilitate classification [22]. Although in our study the images used were obtained by ultrasonographers with substantial experience, we are confident that inexperienced examiners, who tend to perform with lower accuracy, can benefit from using our model. It can raise suspicion of malignancy, allowing high-risk cases to then be referred to specialized centers for appropriate treatment.

In the present study, we decided to exclude borderline cases, as they are rare and hard to diagnose, even by experts [41]. They often require different treatment with fertility-sparing procedures and extensive follow-up since they appear in younger women [42]. Labeling only malignant and benign cases ensured that inadequate data from borderline cases did not affect the system’s performance. The investigation of such cases was thus left outside the scope of this study and deemed as the central topic for our future research.

We observed a pattern where the most frequent benign histopathological types, particularly cystadenomas and endometriomas, were misclassified as malignant by our model. Specifically, 14 cystadenomas and 14 endometriomas were incorrectly classified as malignant. Cystadenomas appear as large and multilocular cysts with mixed echogenicity, which can lead even experts to false-positive results [43]. We noticed that these cystadenomas indeed presented atypical features, leading both the examiners and the CNNs to a false diagnosis. Endometriomas can appear with low-level echogenicity and atypical features, such as papillary projections, also posing challenges for accurate classification [43,44]. The inclusion of women who specifically underwent surgical treatment and the fact that we are an oncology center with many referrals can lead to the assumption that the cases treated are more likely to be complex. Further research is needed to refine these results.

A major consideration during this study was deciding on the validation and testing techniques that would be applied. An issue somewhat overlooked in similar studies is the independence of the test set and the performance of the system on datasets obtained externally [21,24]. Most of the traditional validation methods used while training CNN models suffer from bias, resulting in skewed results in terms of accuracy. Due to the objective difficulties in obtaining data from different examination centers, the data used to train and test the system usually originate from the same facility. To enhance the diversity of our dataset, we included images obtained over a 12-year period by different examiners and ultrasound machines, resulting in a more heterogeneous dataset.

Despite only having data from a single examination center, we also developed a tailored validation technique by modifying the traditional k-fold cross-validation method. The basis behind our approach was ensuring that the training sets for all the folds had high enough variance to train the models on all possible features that can be found in ovarian ultrasound images. To accomplish this, instead of the usual random indexing of the dataset before the training and validation split for each fold, we further divided the dataset into multiple subcategories, labeled with various indicative features. Those included histological type, existence of calipers or not, high or low resolution, etc. We then proceeded to split the dataset into k-folds, ensuring that not only every image appeared at least once in a validation set, but also that each subcategory was adequately represented in each training set.

The second noteworthy differentiation was modifying the validation set for each fold to emulate testing the model with an external dataset. Several modifications were made to the images, mainly consisting of resizing, color corrections, saturation adjustments, and feature repositioning. The goal was to alter the validation dataset as much as possible while maintaining the key features related to the diagnosis of malignancies.

Our study has certain limitations. It was conducted at a single center, which may limit the generalizability of our results to other settings or populations, and it lacked external validation. Moreover, the performance of the model relies on the quality of the ultrasound images, which were obtained by experienced ultrasonographers, potentially limiting its effectiveness with images from less experienced practitioners but also ensuring high-quality and reliable data. To further address these issues, we obtained a heterogenous dataset and employed advanced data augmentation techniques to diversify our data. The use of a modified k-fold cross-validation also enhanced the robustness and reliability of the aggregate model. Lastly, the inclusion of cases with histology reports serves as a gold standard for validation, certifies the accuracy of our ground truth, and strengthens our study’s reliability.

The scheme we designed and developed is meant to be used as a tool in the field of ovarian tumor classification that can be easily used to enhance correct diagnosis and assist non-expert sonographers. Therefore, it can be beneficial for end-users to be able to differentiate and specialize it in detecting or ruling out specific type of tumors, depending on what other systems, tools, and diagnosis techniques it is being paired with. However, future research and validation is needed to generalize the use of the model in different centers since we could not provide external validation to test the model’s performance.

## 5. Conclusions

The results demonstrated the potential of deep learning and AI-assisted image analysis in enhancing the diagnostic process for ovarian tumors. By leveraging pre-trained CNN models, the study achieved high levels of sensitivity and specificity, even surpassing subjective assessment’s sensitivity. The robust performance across diverse k-folds underscores the reliability of the developed classifier. Importantly, the study highlighted the role of automated image analysis in minimizing errors associated with subjective interpretation, thereby improving the diagnostic accuracy. Room for further optimization is there should reducing the time and resources of collecting data become a pressing issue.

## Figures and Tables

**Table 1:** Histopathology of the ovarian masses included.

Histopathology		N (%)
Benign	Cystadenoma (serous, mucinous, and sero-mucinous)	153 (26.2)
	Endometrioma	72 (12.3)
	Mature teratoma	60 (10.3)
	Benign tubal	45 (7.7)
	Corpus luteum	25 (4.2)
	Cystadenofibroma (serous and mucinous)	18 (3.1)
	Thecoma	12 (2.1)
	Fibroma	2 (0.3)
	Other benign	3 (0.5)
	Total benign	390 (66.7)
Malignant	Serous carcinoma	101 (17.3)
	Metastatic	26 (4.4)
	Germ cell malignant tumor	15 (2.6)
	Mucinous carcinoma	15 (2.6)
	Endometrioid carcinoma	12 (2.0)
	Clear cell carcinoma	9 (1.5)
	Sex cord malignant tumor	6 (1)
	Carcinosarcoma	4 (0.7)
	Other malignant tumors	7 (1.2)
	Total malignant	195 (33.3)

**Table 2:** Performance metrics for all the CNNs individually, the aggregate model, and subjective assessment. SA: subjective assessment. AUC: area under the curve. CI: confidence interval.

Metrics	VGG16% (95% CI)	ResNet50% (95% CI)	InceptionNet% (95% CI)	Aggregate% (95% CI)	SA% (95% CI)
Accuracy	87.50 (82.3–91.9)	86.80 (82.6–89.6)	88.90 (83.7–93.5)	90.90 (85.6–93.1)	94.2 (92.3–98.3)
Sensitivity	95.50 (91.1–97.3)	90.20 (86.2–93.2)	88.70 (83.9–91.9)	96.50 (91.2–98.5)	95.90 (93.9–99.1)
Specificity	83.60 (78.7–85.6)	84.90 (79.7–88.1)	88.90 (83.7–93.5)	88.10 (85.1–90.2)	93.60 (88.6–95.9)
AUC	89.50 (84.8–91.9)	87.50 (80.1–90.3)	88.70 (83.9–91.9)	92.20 (90.8–97.1)	-

**Table 3:** Performance metrics for all the CNNs individually, the aggregate model, and subjective assessment for different decision thresholds for the best-performing k-fold.

Threshold	0.1	0.2	0.3	0.4	0.5	0.6	0.7	0.8	0.9
Accuracy	0.932	0.974	0.974	0.949	0.906	0.880	0.855	0.846	0.803
Specificity	0.900	0.963	0.975	0.975	0.988	0.988	1.000	1.000	1.000
Sensitivity	1.000	1.000	0.973	0.892	0.730	0.649	0.541	0.514	0.379
AUC	0.950	0.981	0.974	0.933	0.859	0.818	0.770	0.757	0.689

**Table 4:** Performance metrics for the aggregate model in an average k-fold with a test-set size of 117 cases, for varying weights of pre-trained models. The decision threshold used was 0.2. AUC: area under the curve. FP: false positives. FN: false negatives.

	**VGG16**	**ResNet**	**InceptionNet**	**VGG16**	**ResNet**	**InceptionNet**	**VGG16**	**ResNet**	**InceptionNet**	**VGG16**	**ResNet**	**InceptionNet**
Weights	0.1	0.45	0.45	0.2	0.4	0.4	0.3	0.35	0.35	0.4	0.3	0.3
Accuracy	0.915			0.915			0.915			0.915		
Specificity	0.902			0.89			0.89			0.89		
Sensitivity	0.943			0.943			0.943			0.943		
AUC	0.923			0.931			0.931			0.931		
FP	8			9			9			9		
FN	2			2			2			2		
	**VGG16**	**ResNet**	**InceptionNet**	**VGG16**	**ResNet**	**InceptionNet**	**VGG16**	**ResNet**	**InceptionNet**	**VGG16**	**ResNet**	**InceptionNet**
Weights	0.5	0.25	0.25	0.6	0.2	0.2	0.7	0.15	0.15	0.8	0.1	0.1
Accuracy	0.906			0.897			0.88			0.863		
Specificity	0.878			0.866			0.841			0.829		
Sensitivity	0.971			0.971			0.971			0.943		
AUC	0.925			0.919			0.906			0.886		
FP	10			11			13			14		
FN	1			1			1			2		

**Table 5:** Misclassified cases by the aggregate model and subjective assessment.

Histopathology	Aggregate Model	SA
Benign (Total)	46	26
Cystadenoma	14	10
Endometrioma	14	5
Mature teratoma	6	-
Abscess	3	1
Corpus Luteum	3	1
Hydrosalpinx	2	-
Cystadenofibroma	2	5
Serous Cyst	1	1
Rete ovarii	1	-
Brenner tumor	-	2
Thecoma	-	2
Malignant (Total)	7	8
Serous carcinoma	4	1
Endometrioid carcinoma	2	2
Metastatic	1	2
Ovarian Schwannoma	-	1
Immature teratoma	-	2

## Data Availability

The dataset used in our study is not publicly available due to patients’ privacy and research permissions, but data and results are available upon reasonable request from the corresponding author.
